# Analysis of the CD1 Antigen Presenting System in Humanized SCID Mice

**DOI:** 10.1371/journal.pone.0021701

**Published:** 2011-06-30

**Authors:** Jennifer L. Lockridge, Xiuxu Chen, Ying Zhou, Deepika Rajesh, Drew A. Roenneburg, Subramanya Hegde, Sarah Gerdts, Tan-Yun Cheng, Regan J. Anderson, Gavin F. Painter, D. Branch Moody, William J. Burlingham, Jenny E. Gumperz

**Affiliations:** 1 Department of Medical Microbiology and Immunology, University of Wisconsin School of Medicine and Public Health, Madison, Wisconsin, United States of America; 2 Department of Surgery, University of Wisconsin School of Medicine and Public Health, Madison, Wisconsin, United States of America; 3 Division of Rheumatology, Immunology and Allergy, Brigham and Women's Hospital, Boston, Massachusetts, United States of America; 4 Carbohydrate Chemistry Team, Industrial Research Ltd, Lower Hutt, New Zealand; University Paris Sud, France

## Abstract

CD1 molecules are glycoproteins that present lipids and glycolipids for recognition by T cells. CD1-dependent immune activation has been implicated in a wide range of immune responses, however, our understanding of the role of this pathway in human disease remains limited because of species differences between humans and other mammals: whereas humans express five different CD1 gene products (CD1a, CD1b, CD1c, CD1d, and CD1e), muroid rodents express only one CD1 isoform (CD1d). Here we report that immune deficient mice engrafted with human fetal thymus, liver, and CD34^+^ hematopoietic stem cells develop a functional human CD1 compartment. CD1a, b, c, and d isoforms were highly expressed by human thymocytes, and CD1a^+^ cells with a dendritic morphology were present in the thymic medulla. CD1^+^ cells were also detected in spleen, liver, and lungs. APCs from spleen and liver were capable of presenting bacterial glycolipids to human CD1-restricted T cells. ELISpot analyses of splenocytes demonstrated the presence of CD1-reactive IFN-γ producing cells. CD1d tetramer staining directly identified human iNKT cells in spleen and liver samples from engrafted mice, and injection of the glycolipid antigen α-GalCer resulted in rapid elevation of human IFN-γ and IL-4 levels in the blood indicating that the human iNKT cells are biologically active *in vivo*. Together, these results demonstrate that the human CD1 system is present and functionally competent in this humanized mouse model. Thus, this system provides a new opportunity to study the role of CD1-related immune activation in infections to human-specific pathogens.

## Introduction

CD1 molecules are a family of β2-microglobulin-associated transmembrane glycoproteins that have a structure resembling class I molecules of the major histocompatibility complex (MHC) [1]. There are five different CD1 isoforms, called CD1a, b, c, d and e, each of which is encoded by a distinct gene [2]. Of these, CD1a-d have been shown to present antigens at the cell surface for recognition by T cells, while CD1e is expressed intracellularly and contributes to antigen loading and processing [3]. In contrast to MHC-encoded antigen presenting molecules, CD1 molecules are specialized for binding lipid-containing antigens. Antigen binding to CD1 molecules is thought to occur mainly in intracellular compartments, and since CD1a and CD1b clearly follow different intracellular trafficking routes than CD1c and CD1d, it is thought that different CD1 isoforms may access distinct types of antigens. Additionally, CD1 isoforms are differentially expressed on antigen presenting cell (APC) types, with CD1d expressed broadly by myeloid APCs and B cells, and CD1a, CD1b, and CD1c showing more restricted patterns of expression [1]. As a result of these differences, different CD1 isoforms may carry out divergent antigen presenting functions.

T cells that are restricted by CD1a, CD1b, or CD1c have been implicated in human immune responses to mycobacterial infections (*M. tuberculosis* and *M. leprae*) [4], [5], [6], [7], [8], and have been shown to recognize specific mycobacterial antigens including the lipid mycolic acid [9], glycolipids such as lipoarabinomannan, glucose monomycolate, and mannosyl-β-1-phosphomycoketides [10], [11], [12], [13], as well as lipopeptides such as the didehydroxymycobactins [14]. CD1-restricted T cells that recognized phosphatidylethanolamine derived from pollen appeared to be present in increased frequencies in allergic individuals [15], suggesting that the CD1 system may also contribute to allergic responses. Additionally, the CD1 system may play a role in human autoimmune diseases, since CD1b-restricted T cells recognizing self glycolipids such as gangliosides and sulfatide were isolated from multiple sclerosis patients [16], and autoreactive CD1c-restricted T cells from lupus patients were found to promote IgG secretion by B cells [17]. Thus, these data suggest that CD1a-, CD1b-, and CD1c-restricted T cells are involved in a variety of human diseases. However, progress in understanding the role of these T cells in immune responses *in vivo* has been limited by the lack of a good animal model.

CD1 genes have been detected in all mammalian species analyzed to date, and orthologues have even been identified in birds [18], [19]. However, not all of the CD1 isoforms are expressed in all species, and in some cases CD1 genes have been duplicated, resulting in several variants of the same isoform. Thus, while humans possess one of each of the five CD1 isoforms, this is not the rule. For example, mice and rats have lost the CD1A, B, C, and E genes and have duplicated the CD1D gene, while guinea pigs express multiple variants of CD1B and C, and rabbits and sheep have lost CD1C [18]. Because of these species differences, small animal models that mimic the pattern of CD1 expression found in humans have been lacking, and most *in vivo* analyses have focused on CD1d, which is the isoform that is present in mice.

The CD1d isoform is responsible for selecting a specialized T lymphocyte population called invariant Natural Killer T (iNKT) cells [1]. Murine and human iNKT cells utilize homologous TCRs, show a striking ability to recognize the same microbial glycolipid antigens, and have similar functional properties, including the ability to rapidly produce both Th1 and Th2 cytokines. Analyses of the *in vivo* functions of NKT cells using murine model systems have demonstrated that this subset has a potent ability to modulate immune function, and can markedly impact the outcome of anti-microbial, anti-viral, and anti-tumor responses, as well as ameliorating or preventing the progression of autoimmune diseases. These observations have generated considerable enthusiasm for the possibility that glycolipid antigens that stimulate iNKT cells could be used as therapeutic agents to treat human diseases [20], [21].

However, there are also important differences between humans and mice in regards to the iNKT cell compartment. One of the major differences is that human iNKT cells appear to be present at approximately 100-fold lower frequencies than murine iNKT cells [22], [23], and therefore it is not clear that human iNKT cells will have as potent immunological impacts as those observed in laboratory mice. Another important factor is that murine and human CD1d molecules show differences in intracellular trafficking, which may result in antigen presentation differences [3]. This may be reflected in the differences between murine and human iNKT cells in their requirements for activation by endogenous antigens. Whereas the activation of murine iNKT cells by endogenous ligands requires CD1d molecules to traffic through the endosomal system [24], [25], we have found that human iNKT cells appear to be equivalently activated by wild-type CD1d molecules that undergo endosomal trafficking and mutant CD1d molecules that do not [26]. Moreover, current data suggest that humans lack a glycolipid called isoglobotrihexosylceramide (iGb3) that is thought to endogenously activate murine NKT cells [27], [28]. On the other hand, many human NKT cells recognize an endogenous mammalian lipid called lyso-phosphatidylcholine (LPC) [29], but it is not yet clear whether murine iNKT cells recognize this antigen. Thus, although mice clearly provide an extremely valuable *in vivo* model of iNKT cell function, the successful development of glycolipids as therapeutic agents that activate iNKT cells to modulate human immune responses in specific ways may require a system that permits *in vivo* analysis of the human CD1d pathway.

## Results

### Human hematopoietic cell engraftment

**Figure 1:**
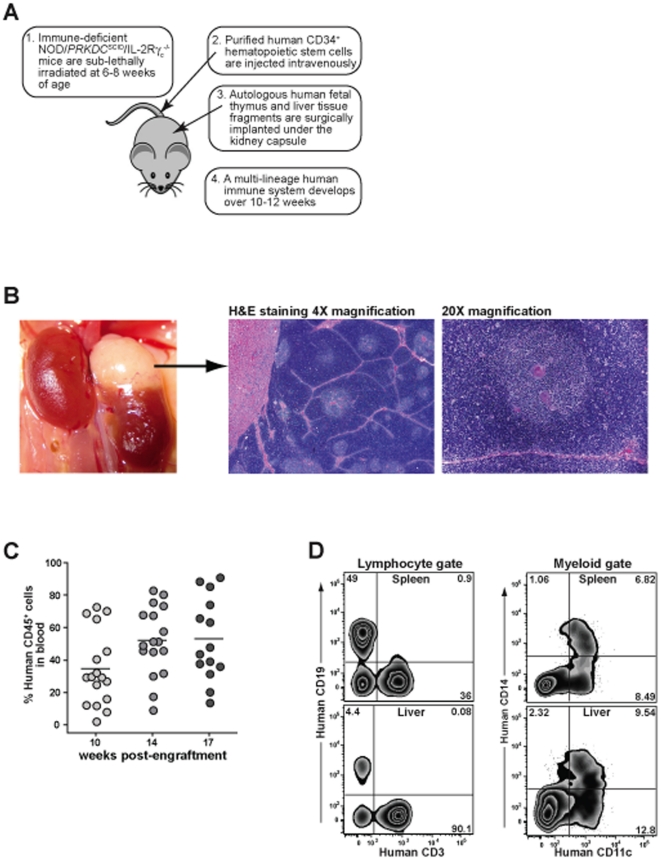
Immune reconstitution of engrafted mice. **A**) Schematic representation of the protocol used to engraft mice with human cells and tissues. **B**) A photograph taken from the abdominal side shows an engrafted human thymic organoid growing on the lateral superior surface of a murine left kidney. Histological analysis of the thymic tissue shows a lobular organization with cortical and medullary areas. The medullary area shown at 20× magnification on the right includes several bodies that closely resemble the Hassall's corpuscles found in human thymus. **C**) Blood samples from engrafted mice were taken at the indicated times and analyzed by flow cytometry to determine the percentage of the total blood leukocytes expressing human CD45. **D**) Flow cytometric analysis of cells isolated from spleen (top panels) and liver (bottom panels) of engrafted mice. The samples were first gated on the human CD45^+^ subset, and then gated by forward and side scatter to focus on lymphocytic cells (left panels) or myeloid cells (right panels).

To generate ‘humanized’ mice we followed an approach that has been described previously [30], in which 6–8 week old NOD/Prkdc^scid^/γ_c_^null^ (NSG) mice are sub-lethally irradiated, then fragments of autologous human fetal liver and fetal thymus are implanted next to each other under the kidney capsule, and concurrently, CD34^+^ hematopoietic stem cells purified from the fetal liver are injected intravenously (Figure 1A). The human CD34^+^ cells colonize the murine bone marrow and undergo hematopoiesis, giving rise to a variety of human leukocyte populations that occupy peripheral lymphoid sites such as the spleen, liver, and lymph nodes. The fragments of human fetal thymic tissue, which are about 1 mm^3^ when they are implanted, expand in size and develop into a vascularized organoid that shows a lobular organization typical of thymus (Figure 1B). In contrast, the human fetal liver fragment becomes undetectable by 8–10 weeks post-engraftment.

We have found that the frequency of human hematopoietic cells (i.e. human CD45^+^) in the blood of the mice increases steadily for about the first 10–12 weeks after engraftment, and thereafter the levels remain comparatively constant [31]. Although there is mouse-to-mouse variation in the degree of human chimerism, blood samples from our humanized mice typically contained 20–70% (mean 53%) human CD45^+^ cells by 12–14 weeks post-engraftment (Figure 1C). Analysis of human cells isolated from spleen or livers of mice 12 or more weeks post-engraftment consistently demonstrated the presence of T cells (CD3^+^), B cells (CD19^+^), macrophages or monocytic cells (CD14^+^), and CD11c^+^ cells that were negative for CD14 (Figure 1D). Thus, we were able to obtain successful reconstitution of multiple lineages of human hematopoietic cells within NSG mice.

### Analysis of the human thymic organoid

**Figure 2:**
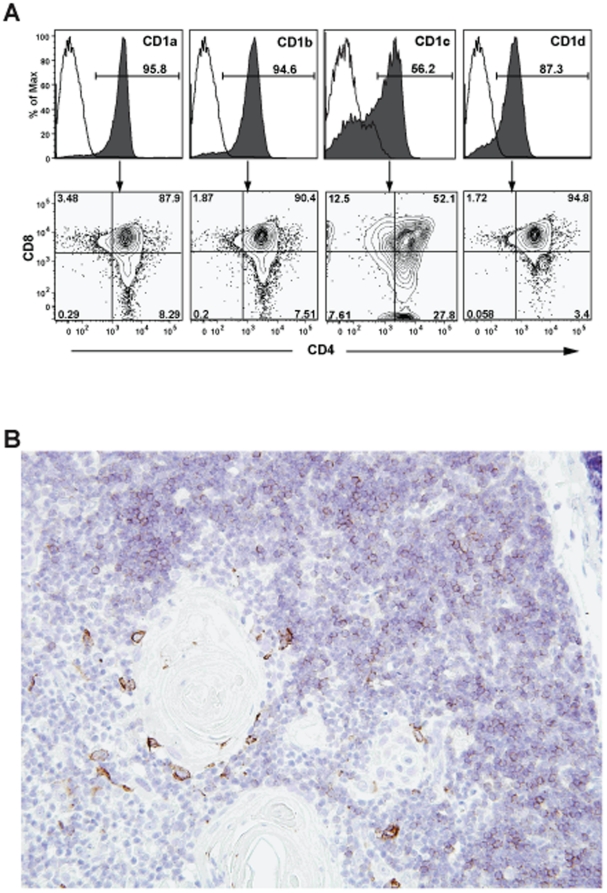
Expression of human CD1 molecules in the engrafted thymic organoid. **A**) Flow cytometric analysis of human CD3^+^ cells from the thymic organoid. Top row: staining with antibodies specific for the indicated CD1 molecules is shown by the filled histograms, open histograms show staining by isotype-matched negative control antibodies. Bottom row: contour plots showing CD4 and CD8 staining of the CD1-positive populations. **B**) Immunohistological analysis of the engrafted thymic organoid at 40× magnification, using an antibody against human CD1a, with DAB chromogenic development (brown color) and hematoxylin counterstaining of nuclei (blue color).

Murine CD1d-restricted NKT cells have been found to be positively selected by CD1d molecules that are expressed on other thymocytes (i.e. developing T cells), and to be negatively selected by thymic dendritic cells [32], [33]. Therefore, we examined the thymic organoids of engrafted (“hu-NSG”) mice for evidence that the development of human CD1-restricted T cells might be supported. Histological analysis of thymic organoid sections stained with hematoxylin and eosin showed densely packed cortical zones surrounding somewhat less densely packed medullary areas (Figure 1B). Towards the center of the medullary areas, eosinophilic concentric circle-like structures were visible that closely resemble the Hassall's corpuscles found in human thymus (Figure 1B). Flow cytometric analysis of cells isolated from the human thymic organoid revealed clear expression of human CD1a, CD1b, CD1c, and CD1d on most CD3^+^ cells (Figure 2A). The CD1^+^ T cells were mainly double positive for the co-receptors CD4 and CD8 (Figure 2A), as is characteristic of cortical thymocytes. Immunohistochemical analysis of the thymic organoid for human CD1a expression revealed positive staining on small round lymphocytic cells packing the outer cortical areas, while most of the small round lymphocytic cells in the inner medullary areas appeared negative for CD1a (Figure 2B). However, there were some larger cells present in the medullary areas that showed intense staining for CD1a, and these tended to be localized near the edges of the Hassall's corpuscles (Figure 2B). These results suggest that that there are two distinct types of CD1^+^ cells within the engrafted human thymic organoid: developing thymocytes localizing mainly to the cortical areas, and larger cells in the medullary areas that may be dendritic cells (DCs). Based on this analysis, we would expect that human CD1-restricted T cells can be both positively and negatively selected in the engrafted thymic organoid.

### Peripheral expression of human CD1 molecules

**Figure 3:**
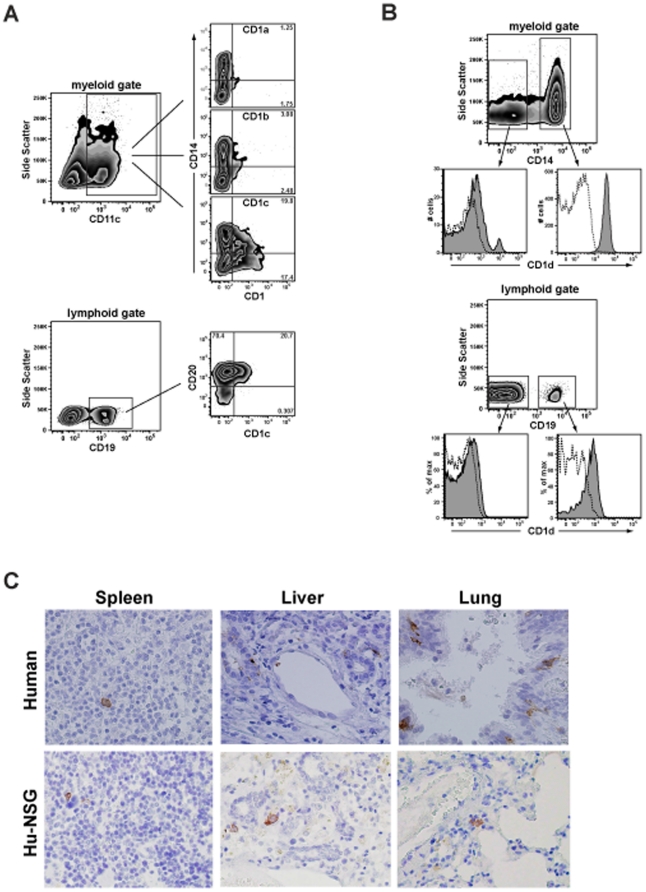
Expression of human CD1 molecules in peripheral tissues. **A and B**) Flow cytometric analysis of splenocytes for CD1a, CD1b,CD1c, and CD1d. The samples were first gated on the human CD45^+^ subset, and then gated by forward and side scatter to focus on myeloid or lymphoid cells. **C**) Histological analysis of CD1a expression in spleen, liver, and lung sections from engrafted mice (bottom row) or human tissue samples (top row). CD1a expression is visualized by DAB staining (brown colored cells). The sections were counterstained with hematoxylin, producing blue staining of cell nuclei. All images are shown at 40× magnification.

We next evaluated the expression of human CD1 molecules in peripheral tissues of engrafted mice. Human CD1a, CD1b, and CD1c molecules are most prominently expressed on myeloid DC lineages, although a fraction of B lymphocytes are typically positive for CD1c. Spleen cells from hu-NSG mice were analyzed by flow cytometry for co-expression of CD1 molecules and lineage markers characteristic of myeloid antigen presenting cells and B cells. Human cells within the myeloid gate that were positive for CD11c (a marker of DCs) showed only very little positive staining for CD1a or CD1b, but a substantial fraction were typically positive for CD1c (Figure 3A). CD1c expression was observed on both CD14^+^ (i.e. monocytes and macrophages) and CD14^−^ myeloid cells (e.g. DCs). Additionally, a substantial fraction of the CD19^+^ lymphocytes typically stained positively for CD1c (Figure 3A). CD1c expression on B cells appeared limited to those that co-expressed CD20, a phenotype that is characteristic of mature B cells.

Human CD1d molecules are constitutively expressed at low cell surface levels by monocytes, macrophages, myeloid DCs, and B lymphocytes [34]. Consistent with this, human CD14^+^ cells in the engrafted mice appeared uniformly positive for CD1d, and a small fraction of the CD14^−^ myeloid cells (possibly myeloid DCs) also expressed high levels of CD1d (Figure 3B). Moreover, B cells in the engrafted mice appeared nearly uniformly positive for CD1d, with very low cell surface expression levels (Figure 3B). These patterns of CD1a, b, c, and d expression closely resemble what we have observed in similar flow cytometric analyses of mononuclear cells freshly isolated from human blood (data not shown).

To further assess the presence of CD1^+^ APCs in peripheral tissues of engrafted mice, we performed immunohistochemical staining for CD1a on sections of spleen, liver, lung, and skin. Some of the hu-NSG spleen sections analyzed showed isolated cells that stained positively for CD1a, and similarly rare positive staining for CD1a was observed in adult human spleen sections (Figure 3C). Multiple cells staining positively for CD1a could be observed in liver sections from the hu-NSG mice and in sections of adult human liver, suggesting that CD1a^+^ APCs might be more abundant in this tissue (Figure 3C). We also observed CD1a^+^ cells in lung sections analyzed from the hu-NSG mice, although they appeared less abundant than those in adult human lung sections (Figure 3C). We were not able to identify CD1a^+^ cells in skin sections from the hu-NSG mice (data not shown), although CD1a^+^ Langerhans cells are typically prominently expressed in human skin. Together, these results indicate that CD1^+^ APCs are present at major sites of immunological activity of engrafted mice, including spleen, liver, and lung, although they may be largely lacking in the skin.

### Presentation of glycolipid antigens

**Figure 4:**
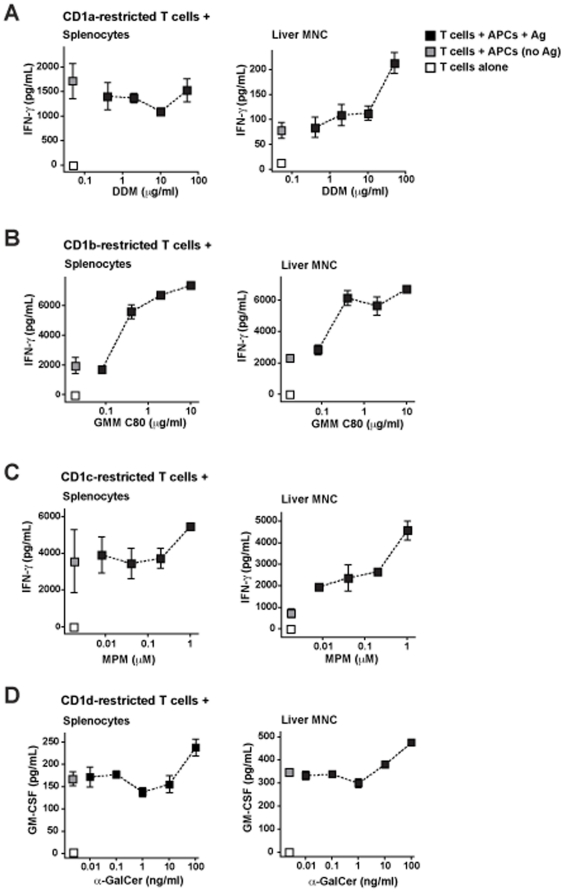
Analysis of CD1-mediated antigen presentation. Mononuclear cells from spleen or liver were used to stimulate cytokine secretion by human CD1-restricted T cell lines in the presence of specific cognate antigens. Black squares show cytokine secretion detected from incubation of human T cells with spleen or liver cells from engrafted mice in the presence of the glycolipid antigen concentrations indicated on the x-axis; grey squares show cytokine secretion detected from T cells incubated with APCs in the absence of glycolipid antigen; and white squares show the results from T cells incubated alone. The squares represent the mean cytokines values from 2–4 replicate analyses with error bars showing standard deviations. **A**) CD1a-mediated antigen presentation was tested using a cell line called CD8-2 and the antigen didehydroxymycobactin (DDM). **B**) CD1b-mediated antigen presentation was tested using a cell line called LDN5 and the antigen glucose monomycolate (GMM). **C**) CD1c-mediated antigen presentation was tested using a cell line called CD8-1 and the antigen mannosylphosphomycoketide (MPM). **D**) CD1d-mediated antigen presentation was tested using an iNKT cell line called J24L.17 and the antigen α-galacosylceramide (α-GalCer). Results are representative of 2–3 independent experiments.

To investigate CD1-mediated antigen presentation by APCs from hu-NSG mice, we isolated cells from spleen or liver and tested their ability to stimulate antigen-dependent cytokine secretion by previously established and characterized human CD1-restricted T cell lines. To test for antigen presentation by CD1a, we used transfected JRT.3 cells expressing a TCR (CD8-2) that has been found to specifically recognize didehydroxymycobactin (DDM) presented by CD1a [14]. We observed marked human IFN-γ production when CD8-2 transfected JRT.3 cells were incubated with spleen cells from an engrafted mouse, however, the cytokine response was not increased by addition of the DDM antigen (Figure 4A, left panel). In contrast, liver mononuclear cells stimulated only modest IFN-γ production from the transfected JRT.3 cells in the absence of DDM, and addition of the antigen resulted in enhanced cytokine secretion (Figure 4A, right panel). Thus, APCs from the liver of hu-NSG mice appeared to be able to perform CD1a-mediated antigen presenting functions, but it was not clear whether splenic APCs shared this ability.

A CD1b-restricted human T cell line called LDN5, which specifically recognizes the mycobacterial lipid glucose monomycolate (GMM) [11], was used to evaluate CD1b-mediated antigen presenting function. Co-incubation of LDN5 cells with splenocytes or liver cells from hu-NSG mice resulted in some IFN-γ production in the absence of added antigen, but there were clear dose-dependent increases in IFN-γ secretion in response to GMM (Figure 4B). Notably, the form of GMM used for these studies contains a long (80 carbon) acyl chain, and has been shown to require internalization into endocytic compartments for loading into CD1b molecules before it can be presented at the cell surface [35]. Thus, these results suggest both spleen and liver contained cells capable of internalizing and presenting a glycolipid antigen via CD1b.

CD1c-mediated antigen presentation was tested using the CD8-1 human T cell line, which recognizes a microbial lipid called mannosylphosphomycoketide (MPM) and also responds to structurally related mammalian lipids called mannosylphosphodolichols [7]. Similar to the results for CD1a-mediated antigen presentation, we found that co-incubation of the CD8-1 cells with hu-NSG splenocytes resulted in marked IFN-γ secretion, and there was little evidence of enhanced responses in the presence of added MPM (Figure 4C, left panel). However, when CD8-1 cells were incubated with liver mononuclear cells from hu-NSG mice there was only modest cytokine production in the absence of added antigen, and clear dose-dependent increases were observed in response to added MPM (Figure 4C, right panel). From these results, it seems that APCs in hu-NSG liver can present added glycolipid antigens via CD1c. However, it is not clear whether the comparatively abundant CD1c^+^ cells we detected in our flow cytometric analyses of hu-NSG spleen samples (see Figure 3A) are also able to present an exogenous glycolipid.

Finally, we used a human invariant NKT cell clone to investigate CD1d-mediated antigen presentation. Human iNKT cells respond to the synthetic glycolipid α-GalCer as a strong TCR agonist [36], but they typically also show detectable cytokine production in response to CD1d^+^ APCs in the absence of added antigens, which is thought to be due to recognition of endogenous CD1d ligands such as lyso-phosphatidylcholine [29]. Similar to the autoreactive responses of human iNKT cells to human CD1d^+^ APCs that we have observed previously, there was clearly detectable cytokine production when human iNKT cell clones were co-incubated with either spleen or liver cells from hu-NSG mice in the absence of added antigens (Figure 4D). The addition of α-GalCer stimulated increased cytokine production from co-cultures of hu-NSG spleen or liver cells with human iNKT cell clones (Figure 4D), indicating it was presented by CD1d^+^ APCs. Notably, the cytokine production was blocked by addition of an antibody that is specific for human CD1d and blocks iNKT cell responses to this antigen presenting molecule, but does not block their responses to murine CD1d (Figures S1 and S2). Together these results suggest that the main route of α-GalCer presentation was through human CD1d molecules. However, it important to note that murine CD1d is not ablated in these mice, and therefore it is possible that CD1d-restricted responses could also be mediated by murine APCs in this system. We also observed enhanced cytokine secretion from the addition of GalGalCer to the co-cultures of human iNKT cells with hu-NSG liver mononuclear cells (Figure S3). GalGalCer is a form of α-GalCer that requires intracellular processing in order to be recognized by iNKT cells [37], and thus this finding indicates that hu-NSG APCs are able to internalize an exogenous glycolipid and trim off excess sugars before presenting it via CD1d.

### Detection and analysis of CD1-restricted T cells

**Figure 5:**
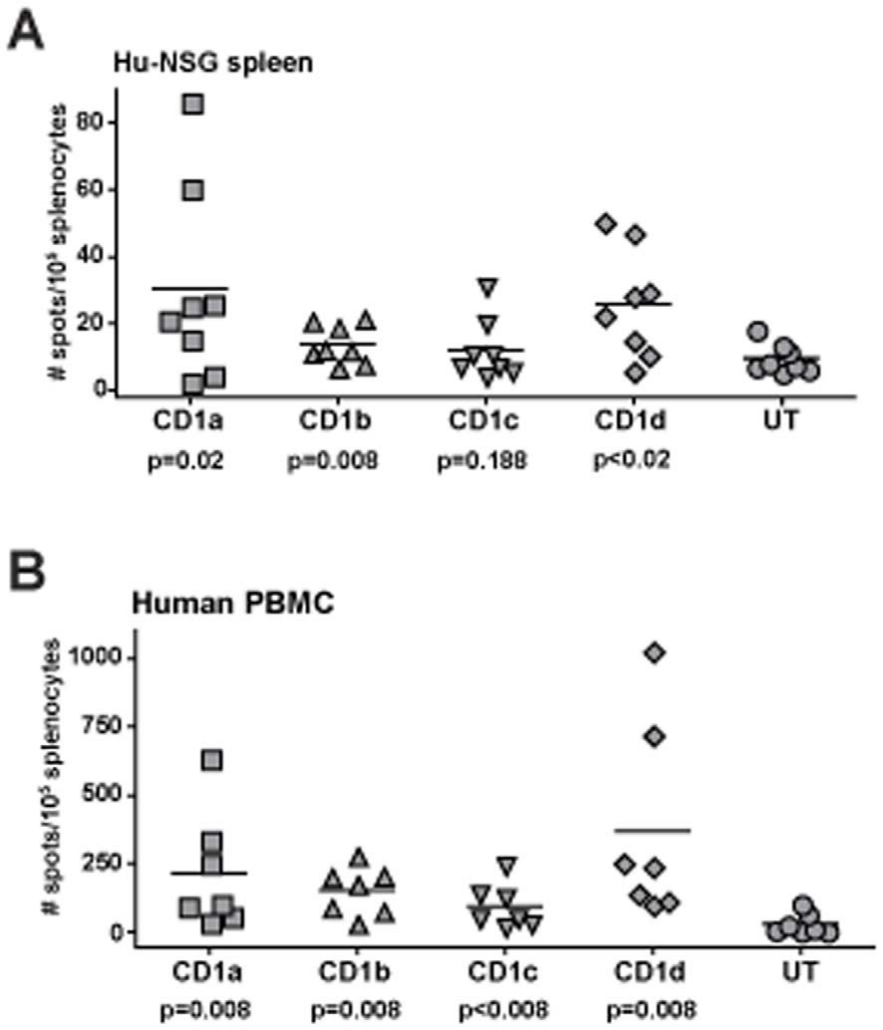
Analysis of CD1-restricted T cell responses. **A**) Splenocytes from engrafted mice were combined with human K562 antigen presenting cells transfected with the indicated CD1 molecules, or with the untransfected K562 parental cell line (“UT”), and the frequencies of IFN-γ producing cells were estimated by ELISpot analysis. Each symbol represents the mean number of spots from 3 or 4 replicate analyses of the splenocytes from one engrafted mouse. The data were statistically analyzed using a one-tailed Wilcoxon paired t-test, yielding the p values shown below the x-axis labels for each CD1 transfectant compared to the untransfected parental cell line. **B**) Results from a similar analysis using peripheral blood mononuclear cells (PBMC) purified from healthy adult human donors.

Previous analyses have indicated that a significant fraction of human CD1a-, CD1b-, and CD1c-restricted T cells resemble iNKT cells in that they are able to become activated in a CD1-dependent manner in the absence of foreign antigens [38], [39], [40], [41], [42]. To test for the presence of such T cells in hu-NSG mice, we performed IFN-γ ELISpot analyses using CD1-transfected K562 myelo-monocytic cells as APCs. Hu-NSG splenocytes were incubated in ELISpot wells with CD1a-, CD1b-, CD1c-, or CD1d-transfected K562 cells, or with untransfected (CD1-negative) K562 cells. K562 cells were used for these analyses because they are negative for MHC class I and II molecules, and therefore these APCs would be unlikely to stimulate an allo-response by MHC-restricted T cells [42]. The untransfected K562 cells stimulated IFN-γ spot numbers that were close to background levels (i.e. splenocytes without any added APCs). In contrast, the CD1a-, CD1b-, and CD1d-transfected K562 cells consistently stimulated greater numbers of spots (Figure 5A). In some cases, the CD1c-transfected K562 cells also stimulated elevated numbers of spots, but this was not sufficiently consistent to attain statistical significance (Figure 5A). Peripheral blood mononuclear cells isolated from healthy adult human donors showed a similar pattern of ELISpot results in response to the CD1-transfected K562 cells (Figure 5B). Moreover, we observed similar responses to the CD1-transfected K562 cells by liver mononuclear cells from engrafted mice (data not shown). Thus, the hu-NSG mice appear to possess IFN-γ producing lymphocytes that respond to CD1 molecules loaded with cellular ligands.

**Figure 6:**
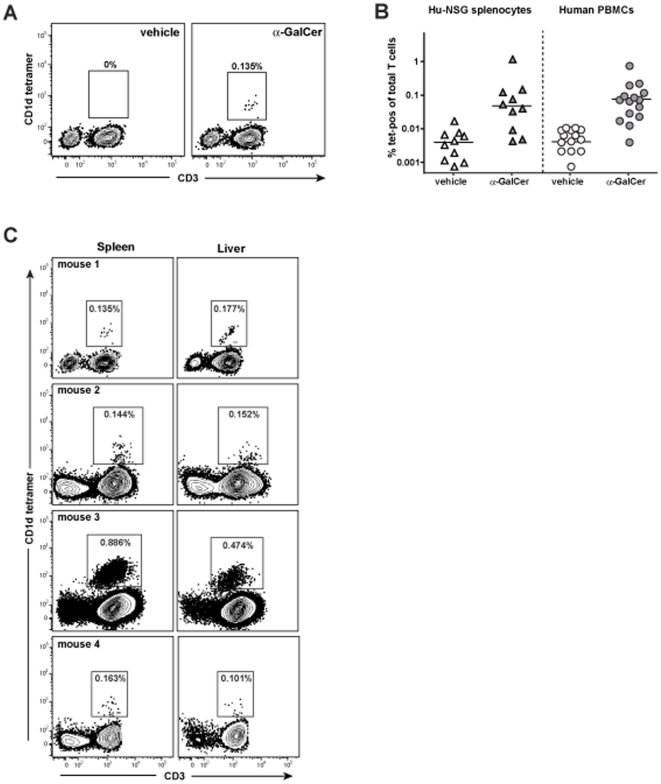
Detection of iNKT cells. **A**) Flow cytometric analysis of splenocytes from an engrafted mouse using fluorescently labeled human CD1d tetramer (y-axis) and an antibody against human CD3 (x-axis). The left plot shows staining using vehicle treated CD1d tetramer, and the right plot shows α-GalCer loaded CD1d tetramer. **B**) Percentages of CD1d tetramer-positive cells detected from splenocytes of 10 engrafted mice, compared to PBMCs from 15 healthy adult human donors. The medians for each data group are as follows: spleen cells from hu-NSG mice, tetramer+vehicle = 0.0039%, tetramer+α-GalCer = 0.048%; human PBMCs, CD1d tetramer+vehicle = 0.0041%, CD1d tetramer+α-GalCer = 0.0745%. **C**) Analysis of the splenocytes and liver mononuclear cells from 4 engrafted mice for human cells stained by CD3 and α-GalCer loaded CD1d tetramer.

In our analyses of CD1-mediated antigen presentation by splenocytes from hu-NSG mice, we did not observe significantly increased IFN-γ secretion by splenocytes that were treated with α-GalCer compared to untreated splenocytes (Figure S4), raising the question of whether iNKT cells are present in the engrafted mice. Therefore, to specifically investigate the frequency of CD1d-restricted iNKT cells, we used CD1d tetramers loaded with α-GalCer. By flow cytometric analysis we observed T cells that stained specifically with α-GalCer loaded CD1d tetramer, whereas there was little or no staining using a vehicle treated control tetramer (Figure 6A). The frequency of CD1d tetramer^+^ T cells varied substantially among the hu-NSG mice tested, but the mean and the range appeared similar to that observed in PBMC samples of healthy human donors (Figure 6B). Notably, whereas commonly used laboratory strains of mice have substantially elevated frequencies of iNKT cells in the liver compared to the spleen [43], the hu-NSG mice typically showed similar frequencies of tetramer positive cells in the spleen and liver (Figure 6C). This lack of iNKT cell enrichment in the liver is similar to what has been observed in previous analyses of primary human liver samples [44], [45], [46].

**Figure 7:**
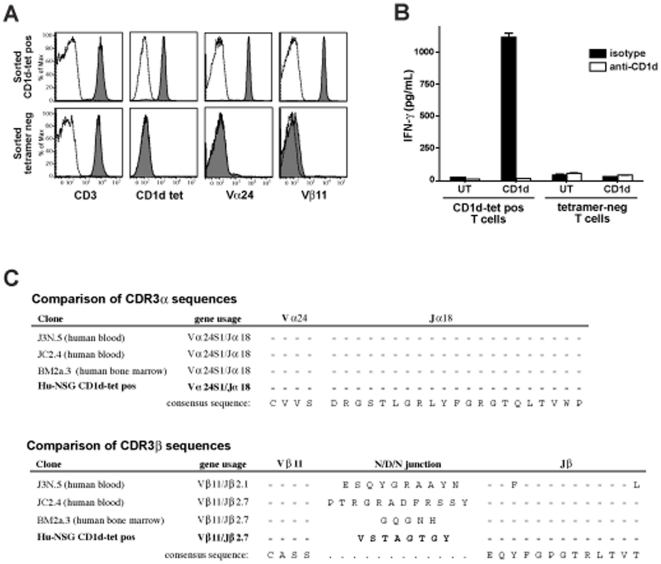
Analysis of hu-NSG NKT cells. CD1d tetramer-positive and -negative T cells were flow cytometrically sorted from an engrafted mouse and expanded *in vitro*. **A**) Flow cytometric analysis of the expanded cells (tetramer-positive top row, tetramer-negative bottom row). Filled histograms show staining with fluorescently labeled anti-CD3, α-GalCer loaded human CD1d tetramer, anti-Vα24, and anti-Vβ11 antibodies, respectively. Open histograms show staining with vehicle treated CD1d tetramer or isotype-matched negative control antibodies. **B**) Cytokine secretion by the tetramer-positive and -negative cell lines in response to CD1d-transfected or untransfected (UT) APCs in the presence of α-GalCer. Open bars indicate assays that were performed in the presence of an anti-CD1d blocking antibody, and filled bars show assays performed in the presence of an isotype matched negative control antibody. **C**) Predicted amino acid sequences of the junctional regions of the TCRα and β chains from hu-NSG tetramer-positive T cells compared to those of NKT cell clones previously derived from the human tissues shown in parentheses. Dashes indicate identity with the consensus sequences shown at the bottom.

To investigate the TCR usage of CD1d-restricted NKT cells from hu-NSG mice, we sorted T cells stained by the α-GalCer loaded CD1d tetramer and expanded them *in vitro*. CD1d tetramer-positive and -negative T cells were sorted separately, and expanded by exposure to PHA and irradiated allogeneic human feeder cells in the presence of recombinant IL-2. Flow cytometric analysis of the expanded cells indicated that the CD1d tetramer-positive cells used Vα24 and Vβ11 TCR chains, whereas the tetramer-negative T cell line sorted in parallel showed little or no staining for these TCR chains (Figure 7A). The CD1d tetramer-positive cells secreted cytokines in response to CD1d-transfected APCs that were pulsed with α-GalCer, but did not respond to the untransfected parental cell line, and the cytokine secretion was blocked by an anti-CD1d antibody (Figure 7B). DNA sequence analysis confirmed that the TCRα chain of the CD1d tetramer-positive cells was Vα24, and demonstrated that it was rearranged to Jα18 using the same canonical rearrangement found in human iNKT cells (Figure 7C, top). Analysis of the TCRβ chain identified a single dominant sequence consisting of Vβ11 rearranged to Jβ2.7, with a unique sequence at the V-J junctional region that is suggestive of N-region diversification (Figure 7C, bottom). Thus, the hu-NSG mice possess human CD1d-restricted T cells that utilize the canonical TCRa chain paired with Vβ11, and that recognize α-GalCer presented by CD1d.

### Activation of NKT cells *in vivo*

**Figure 8:**
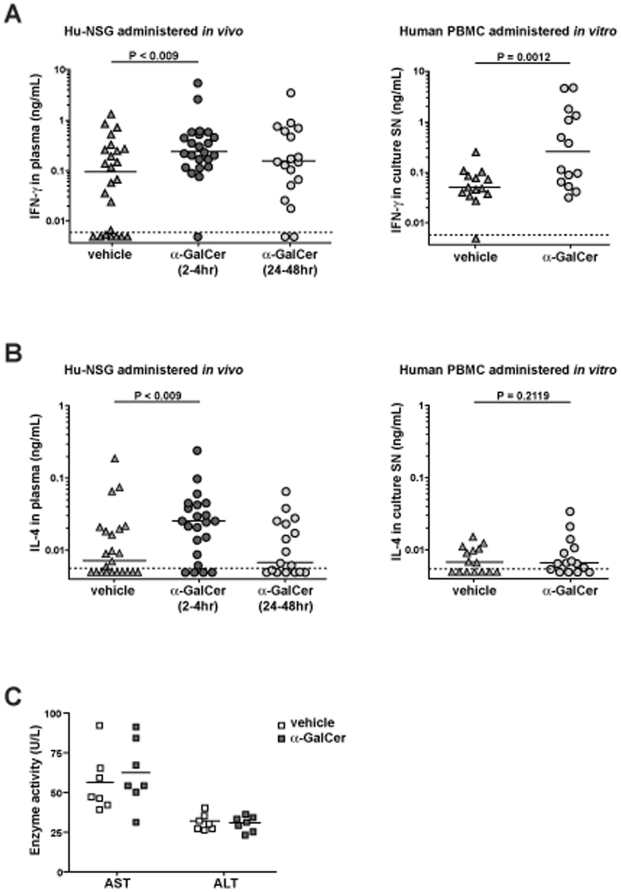
Activation of NKT cells *in vivo*. **A**) The plot on the left shows results from engrafted mice that were injected intraperitoneally with 3 µg α-GalCer or with an equivalent amount of vehicle alone. Blood samples were collected at 2–4 and 24–48 hours after injection and analyzed for human IFN-γ by ELISA. Each symbol represents the mean of 3 replicates. The P value shown on the plot was calculated using a one-tailed unpaired t test. The plot on the right shows results from human PBMC samples that were incubated in vitro with α-GalCer or vehicle alone. Supernatants were collected after 48 hours and analyzed for IFN-γ by ELISA. The P value shown on the right plot was calculated using a one-tailed paired t test. **B**) Results from the same experiments analyzed for human IL-4 by ELISA. **C**) Heparinized plasma samples taken at 48-hours post injection were analyzed for enzyme activity of the liver transaminases AST and ALT, which serve as biomarkers of liver injury. The plot shows the enzyme activities detected from samples from mice that were treated with vehicle (open squares) or with α-GalCer (filled squares). There are no significant differences between the groups.

Injection of α-GalCer into common strains of laboratory mice results in rapidly detectable immunological activation that is characterized by elevated levels of IFN-γ and IL-4 in the serum that correlate with the frequency of iNKT cells [43]. To test the effects of *in vivo* administration of α-GalCer in this model, hu-NSG mice were injected intraperitoneally with 3 µg of α-GalCer or an equivalent amount of vehicle alone. Blood samples were taken from the mice at 2–4 or 24–48 hours post-injection, and the plasma was analyzed for human IFN-γ and IL-4 by ELISA. The median IFN-γ concentration of samples from the vehicle treated mice was 95 pg/ml, with about a third of the samples having IFN-γ values that were near or below the limit of detection for the ELISA (Figure 8A, left plot). In contrast, only one sample from the 2–4 hour post α-GalCer injection time point was below the limit of detection of the ELISA, and the median IFN-γ concentration was 240 pg/ml (Figure 8A, left plot). Although the amount of IFN-γ from the α-GalCer treated mice was only 2–3 fold greater than that of the vehicle treated mice, a Mann-Whitney test indicated that the difference is statistically significant (p<0.009). The median IFN-γ detected from the 24–48 hours post α-GalCer injection samples was 155 pg/ml, and statistical analyses indicated that this was not significantly elevated compared to the vehicle treated mice (Figure 8A, left plot). By comparison, when we incubated samples of 0.5 million human PBMCs *in vitro* in 0.2 mL of culture medium containing either α-GalCer or vehicle, we detected a median IFN-γ concentration of 50 pg/ml for the vehicle treated and 255 pg/ml for the α-GalCer treated samples (Figure 8A, right plot). Interestingly, whereas we were not able to detect any increased IL-4 in the culture supernatants from human PBMCs incubated *in vitro* with α-GalCer compared to vehicle (Figure 8B, right plot), we observed a significant increase in the amount of IL-4 detected from blood samples of hu-NSG mice taken at 2–4 hours post α-GalCer injection compared to those of vehicle treated mice (Figure 8B, left plot). These results demonstrate that treatment with α-GalCer induces a rapid biological response *in vivo* in the hu-NSG mice, with systemic cytokine levels apparently becoming elevated in the first few hours after treatment and then receding to baseline levels within 24–48 hours. Analysis of plasma samples from α-GalCer or vehicle treated mice that were taken at 48-hours post-injection showed no difference in levels of the liver transaminases AST and ALT (Figure 8C), suggesting that the liver injury that has been observed in laboratory mice following α-GalCer treatment is much less significant in this model [47].

## Discussion

Great progress has been made using murine model systems to understand the development and function of CD1d-restricted iNKT cells, however, it remains unclear whether human iNKT cells are identical to their murine counterparts. Additionally, little is known about the development and *in vivo* function of T cells that are restricted by other CD1 molecules. The analyses presented here demonstrate that immunodeficient mice that are transplanted with human tissues (hematopoietic stem cells, fetal thymus, and fetal liver) develop a functional human CD1 antigen presenting system in the periphery and acquire human iNKT cells with a canonical TCR rearrangement. Additionally, our findings suggest that the development of other human CD1-restricted T cells is supported. Thus, these mice provide a very promising model system for further investigating specific aspects of the development and functions of human CD1-restricted T cells *in vivo*.

In this model system NSG mice are engrafted with human tissues when they are six to eight weeks old. At this age the murine thymus appears to have atrophied and disappeared (presumably because the mice do not produce their own T cells). In any case, we are not able to detect a murine thymus even after engraftment of the human hematopoietic stem cells (data not shown). Therefore, it is not clear whether any of the developing human T cells in this model are selected on murine antigen presenting molecules. However, it is clear that the transplanted human fetal thymic tissue increases in size and persists as a long-lasting thymic organoid. We show here for the first time that there is prominent expression of CD1a, CD1b, CD1c, and CD1d molecules by human T cells in this thymic organoid. In our histological analyses, we observed that there were two distinct CD1a^+^ cell types in the thymic organoid: abundant small, round lymphocytic cells that appear to be cortical thymocytes, and less abundant, larger cells in the medullary area that may be DCs. An interesting possibility is that these two populations are involved in different aspects of thymic selection of CD1-restricted T cells. Whereas MHC-restricted T cells are positively selected by MHC molecules expressed on thymic epithelial cells in the thymic cortex, murine CD1d-restricted NKT cells have been shown to be positively selected in the thymic cortex by CD4 and CD8 double positive thymocytes [32]. Thus, we would expect that the CD1^+^ cortical thymocytes present in the human thymic organoid might mediate positive selection of CD1a-, CD1b-, CD1c-, or CD1d-restricted human T cells. Moreover, since negative selection of murine NKT cells appears to be mediated by DCs of hematopoietic origin [33], we would hypothesize that the CD1a^+^ DC-like cells we observed in the medullary regions of the human thymic organoid might play a role in negative selection.

We also found expression of CD1 molecules by human cells in peripheral tissues of engrafted mice. CD1c and CD1d were clearly detectable on myeloid cell types that were present in the spleen (see Figure 3) and in the liver (data not shown), and these CD1 molecules were also expressed by B lymphocytes (Figure 3). Splenocytes expressing CD1a or CD1b appeared infrequent by flow cytometric analysis, but by histological analysis we were able to detect rare cells that stained positively for CD1a in the spleen, as well as somewhat more abundant CD1a^+^ cells in liver and lung sections. Functional analyses demonstrated that liver mononuclear cells from engrafted mice were able present specific glycolipid antigens to CD1a-, CD1b-, CD1c-, or CD1d-restricted human T cells. Spleen cells were able to present glycolipid antigens to CD1b- and CD1d-restricted T cells, but the CD1a- and CD1c-restricted T cells we tested showed a high response to the splenocytes alone (without added antigen) and it was difficult to see enhanced activation in response to added antigen. Thus, the splenic APCs may stimulate responses by some CD1-resticted T cells that are not dependent on the addition of foreign antigens. It is not clear whether this is due to presentation of endogenous antigens by the relevant CD1 molecules, or whether this is due to some sort of cross-reactivity by the human T cell lines tested here for murine antigen presenting molecules. We favor the first explanation, since the antigen-independent responses were not apparent using the liver APCs (which would also be expected to contain cells expressing murine antigen presenting molecules), however, further studies will be required to determine whether xeno-presentation is a factor.

Our ELISpot analyses revealed that there are cells in the spleens of engrafted mice that produce IFN-γ in a CD1-dependent manner without requiring addition of foreign glycolipids, suggesting the presence of CD1-restricted T cells that respond to endogenous antigens. This is similar to previous observations of CD1-restricted cells from human tissue samples that are not dependent on the addition of foreign antigens [38], [39], [40], [41], [42]. Flow cytometric analyses using human CD1d tetramers revealed a T cell population that was specifically stained by α-GalCer loaded tetramer, and analysis of a cell line derived from the tetramer-stained population demonstrated the use of a canonically rearranged TCR. Whereas murine iNKT cells comprise about 1–5% of splenic T cells and 10–30% in the liver, the iNKT cell subset generally comprised about 0.1% of the total human T cells in the spleens of the engrafted mice, and the frequency did not appear to be enriched in the liver. The iNKT cell frequencies we observed in the hu-NSG mice and their lack of enrichment in the liver are consistent with iNKT cell frequencies observed in previous analyses of human tissues [22], [44], [45], [46], and this therefore appears to constitute an important difference between humans and commonly used strains of laboratory mice. Given the differences in iNKT cell frequency between the hu-NSG mice and laboratory strains, it is perhaps not surprising that injection of α-GalCer resulted in only modest increases in IFN-γ and IL-4 levels in the blood of engrafted mice, and did not appear to produce elevated levels of transaminase enzymes that are indicative of damage to the liver. Nevertheless, our data indicate that there is a rapid cytokine response to α-GalCer in vivo in the engrafted mice, which suggests that these mice will be a valuable tool for investigating the impact of glycolipid antigens in a human immune context.

The CD1 system has a number of key advantages from the perspective of developing new methods of therapeutic immune modulation: the antigens utilized by the CD1 system are highly conserved molecules that may reduce the chances of pathogen escape through mutation, also there is very little allelic polymorphism in the human CD1 system, and finally, CD1-restricted T cells seem to temporally bridge the innate and adaptive immune systems during immune activation and therefore they may occupy a very powerful functional niche. Although humanized mouse models are not without concerns, for example, batch to batch variations, heterogeneity of engraftment within batches, and the possibility of artifacts due to the chimeric nature of the immune system, our results suggest that the hu-NSG mouse model described here will be an extremely useful tool for studying the thymic development and peripheral function of human CD1-restricted T cells *in vivo*. Moreover these mice provide a highly novel opportunity to study the role of the CD1 system *in vivo* during immune responses to human specific pathogens such as Epstein-Barr virus, HIV, and Dengue.

## Materials and Methods

### Generation of humanized mice

Research involving mice was performed in accordance with a protocol that was approved by the University of Wisconsin's Animal Care and Use Committee, and in accordance with a protocol approved by the University of Wisconsin's Institutional Review Board. NOD.Cg-*Prkdc^scid^ IL2rg^tm1Wjl^*/SzJ mice (abbreviated as NOD/SCID/γ_c_^null^ or “NSG” mice) were obtained from Jackson Laboratory (stock #005557). At 6–8 weeks old, mice were conditioned with sub-lethal (2.5 Gy) whole-body irradiation, and within 4 hours were surgically implanted under the kidney capsule with fragments (approximately 1 mm^3^) of human fetal thymus and liver (14–22 weeks gestation). Immediately following this surgery, the mice were given an intravenous injection (4×10^5^/mouse) of CD34^+^ cells isolated from autologous fetal liver tissue that was processed and stored in X-vivo 15 serum free culture medium (Cambrex Biosciences Walkersville, MD). The CD34^+^ cells were prepared by gently disrupting the liver tissue by cutting it into small pieces and repeatedly pipetting up and down, then filtering the resulting cell suspension through a 70 µm mesh, and purifying the mononuclear cells by Ficoll-Paque density gradient separation (GE Healthsciences). CD34^+^ cells were then isolated by magnetic sorting (Miltenyi Biotec, Auburn, CA). The purity of the injected CD34^+^ cells was determined by flow cytometric analysis to be at least 80–90%, with less than 0.5% contamination of CD3^+^ cells. The drinking water for the mice was supplemented with 0.17–0.25 mg/ml Enrofloxacin (Bayer Healthcare, KS) for 10 days post surgery to prevent infections.

### Flow cytometric analysis

Antibodies used to detect specific human markers were as follows: pan CD45 (clone HI30); CD4 (clone RPA-T4 or OKT4); CD8α (clone RPA-T8); CD14 (clone M5E2); CD19 (clone HIB19); CD11c (clone S-HCL-3); CD123 (clone 9F5); pan HLA class I (clone W6/32); pan HLA class II (clone LN3); CD1a (clone OKT6); CD1b (clone BCD1b3.1); CD1c (clone BDCA-1); CD1d (clone CD1d42); CD3 (clone SPVT-3b); Vα24 (clone C15B12); Vβ11 (clone C21D2). Negative control antibodies were clone P3 (IgG1) and clone UPC10 (IgG2a). For flow cytometric analysis, tissues (i.e. bone marrow, spleen, liver, blood, thymic organoid) were collected from mice 12–20 weeks after implantation of human cells. Human tissue samples used as controls (i.e. blood and spleen) were collected in accordance with a protocol approved by the University of Wisconsin Minimal Risk IRB. Single cell suspensions were prepared from solid tissues by gentle homogenization followed by filtration through a 0.45 µm strainer. Samples were subject to ACK lysis to remove red blood cells, or mononuclear cells were purified by density gradient centrifugation. The samples were blocked with human and murine serum, and then incubated on ice for 30 minutes with 10 µg/ml specific antibodies in FACS buffer (1 mg/ml PBS/BSA) containing 10 µg/ml 4′, 6′-diamidino-2-phenylindole (DAPI) or propidium iodide (PI). The samples were washed with FACS buffer, then resuspended and analyzed on a Becton Dickenson LSRII or a FACSCalibur flow cytometer. Data were analyzed using Flowjo software (Tree Star, Inc., Ashland, OR).

### Histologic analysis

Tissues were fixed in 10% buffered formalin and embedded in paraffin. Tissue sections from liver, spleen, lung, lymph node and thymic organoid were de-paraffinized and rehydrated with water for hematoxylin and eosin (H&E) staining or immunohistochemistry. Antigen retrieval was performed using rodent decloaker (Biocare Medical), followed by incubation with a mouse monoclonal against human CD1a (clone CD1a007, Biocare Medical). Antibody labeling was detected and visualized by the Mouse-on-Mouse Horseradish peroxidase (HRP)-Polymer Kit with diamionbenzidine (DAB) development (Biocare Medical).

### Antigen presentation analyses

Spleen and liver tissues were collected from mice 12–20 weeks after implantation of human cells, and single cell suspensions were prepared. The samples were tested for stimulation of human CD1-restricted cell lines in the presence or absence of purified or synthetic lipid antigens. Assays were carried out in 96-well round bottom plates in 200 µl culture medium (RPMI 1640, 10% Bovine Calf Serum, 2 mM L-glutamine, and 100 µg/ml each of penicillin and streptomycin), using 5×10^4^/well hu-NSG cells and an equal number of human T cells. After 16 hours incubation at 37°C in a 5% CO_2_ incubator, culture supernatants were collected and tested for cytokine concentration using commercially available ELISA reagents. Human T cell lines used were as follows: CD8-2, a CD1a-restricted T cell line [48]; LDN5, a CD1b-restricted T cell line [11]; CD8-1, a CD1c-restricted T cell line [48]; and J24L.17, J3N.5, or Jc2.4, human CD1d-restricted NKT cell clones, [36], [49]. Human T cell lines were cultured in T cell medium (RPMI 1640 medium, 2 mM L-glutamine, 100 µg/ml each of penicillin and streptomycin, 10% FBS, 5% bovine calf serum, 5% human AB serum from Gemini Bio-Products, and 400 U/ml recombinant human IL-2 from Chiron), with periodic re-stimulation by irradiated allogeneic feeder cells and PHA.

### Lipid antigens

For stimulation of the CD1a-restricted CD8-2 line, a fraction enriched for the lipopeptide didehydroxymycobactin (DDM) was purified from *M. tuberculosis* [14]. For stimulation of the CD1b-restricted LDN5 line, a long chain form of glucose monomycolate (GMM C80) was purified from *M. phlei* [11]. For stimulation of the CD1c-restricted CD8-1 line, a synthetic preparation of mannosyl-β1-phosphomycoketide (MPM C32) was used [50]. For *in vitro* analyses of CD1d-restricted NKT cells, α-GalCer was prepared as described [51]. CD1a-, CD1b-, and CD1c-restricted T cell lipid antigens were stored in organic solvents at −20°C, and prior to use were transferred to glass vials and dried down under nitrogen gas, then resuspended in culture medium and sonicated in a heated water bath. α-GalCer was dissolved in DMSO at 100 µg/ml, and this solution was sonicated then diluted into culture medium prior to use. For *in vivo* injection, α-GalCer was synthesized by coupling 2,3,4,6-tetra-(*O*-trimethylsilyl)-D-galactosyl iodide with a sphingosine acceptor containing azide functionality, and the resulting compound was modified by azide-reduction, *N*-acylation and deprotection of benzyl-protected hydroxyl groups. The α-GalCer was dissolved in a vehicle solution containing 0.5% Tween 20, 5.7% sucrose, and 0.75% histidine.

### ELISpot analyses of CD1-restricted T cell frequency

Mononuclear cells were prepared from hu-NSG spleen or liver, or from human peripheral blood. Human blood was collected with written consent from volunteer donors, in accordance with a protocol approved by the University of Wisconsin's Minimal Risk Institutional Review Board. The samples were incubated with untransfected human K562 myelomonocytic cells [52] or with K562 cells that were transfected with human CD1a, CD1b, CD1c, or CD1d, at a 1∶1 ratio (200,000 cells per well total) in serum-free medium (CELLect medium, MP Biomedicals, Inc.) in 96-well PVDF membrane plates (Whatman) coated with anti-human IFN-γ mAb (clone NIB42 from Biolegend). The cells were incubated for 48 hr at 37°C and 5% CO_2_. Secreted IFN-γ was detected using biotinylated anti-human IFN-γ mAb (clone M701B from Thermo Scientific), and revealed by development with streptavidin-alkaline phosphatase and BCIP/NBT chromogenic substrate. Spots were quantitated using AID 5.0 software.

### Generation of CD1d-restricted T cell line

Hu-NSG spleen was sterilely harvested and mononuclear cells were purified by density gradient centrifugation. The cells were then stained with sterile fluorescently labeled antibodies against human CD3 and CD19, and with human CD1d tetramer loaded with α-GalCer. Between 50 and 75 tetramer-positive human T cells were flow cytometrically sorted into a well of a 96-well tissue culture plate and cultured with irradiated allogeneic human PBMCs in RPMI 1640 supplemented with 2 mM L-glutamine, 100 µg/ml penicillin and streptomycin, 10% fetal bovine serum (Hyclone), 5% bovine calf serum (Hyclone), 3% human AB serum (Atlanta Biologicals), PHA (Sigma) and 400 U/ml recombinant human IL-2 (Chiron).

### TCR sequence analysis

Total RNA was prepared from approximately 1–5×10^6^ CD1d-restricted T cells using Trizol (Invitrogen), and 1 µg was used for preparation of cDNA using commercially available reagents (Roche). DNA sequences spanning the junctional regions of the TCRα and β chains were amplified by PCR using forward primers that sit down in the Vα24 or Vβ11 gene segments (5′-AAGATACTGGGAGAGGTCCTGTTTC-3′ or 5′-CCAGGAATGGAACTACACCTCATC-3′, respectively), and reverse primers that sit down in the TCRα or β constant domains (5′-GAATAATGCTGTTGTTGAAGGCG-3′ or 5′-TTGACAGCGGAAGTGGTTGC-3′, respectively). The resulting PCR products were purified by spin column or agarose gel extraction (QIAquick from QIAGEN), and were sequenced using the same primers that were used for amplification.

## Supporting Information

Figure S1 Cytokine production from previously established human iNKT cell clones (Jc25., Jc2.8, JJ2.7) in response to spleen cells from an engrafted mouse treated with α-GalCer is blocked by addition of the CD1d42 anti-human CD1d monoclonal antibody, but not by an isotype-matched negative control antibody.
(TIF)

Figure S2 The CD1d42 antibody blocks iNKT cell responses to human but not murine CD1d. Untransfected P815 cells (no CD1d), P815 cells transfected with murine CD1d, and P815 cells transfected with human CD1d were treated with α-GalCer and incubated with a human iNKT cell clone (Jc2.5) in the presence of the anti-CD1d antibody CD1d42 (black bars) or an isotype control mAb (grey bars).
(TIF)

Figure S3 CD1d-mediated antigen presentation by liver cells from engrafted mice was tested using the human iNKT cell clone J24L.17 and a glycolipid called GalGalCer, which requires glycosidic cleavage of the terminal galactose sugar in order to be recognized by iNKT cells.
(TIF)

Figure S4 Splenocytes from an engrafted mouse show no significant responses to antigens used for evaluation of CD1-mediated antigen presentation to human T cell lines.
(TIF)
