# Supplementary figures and images for: Analysis of the CD1 Antigen Presenting System in Humanized SCID Mice
Source: PLoS One. 2011 Jun 30;6(6):e21701. doi: 10.1371/journal.pone.0021701 (PMC3128084; doi:10.1371/journal.pone.0021701)

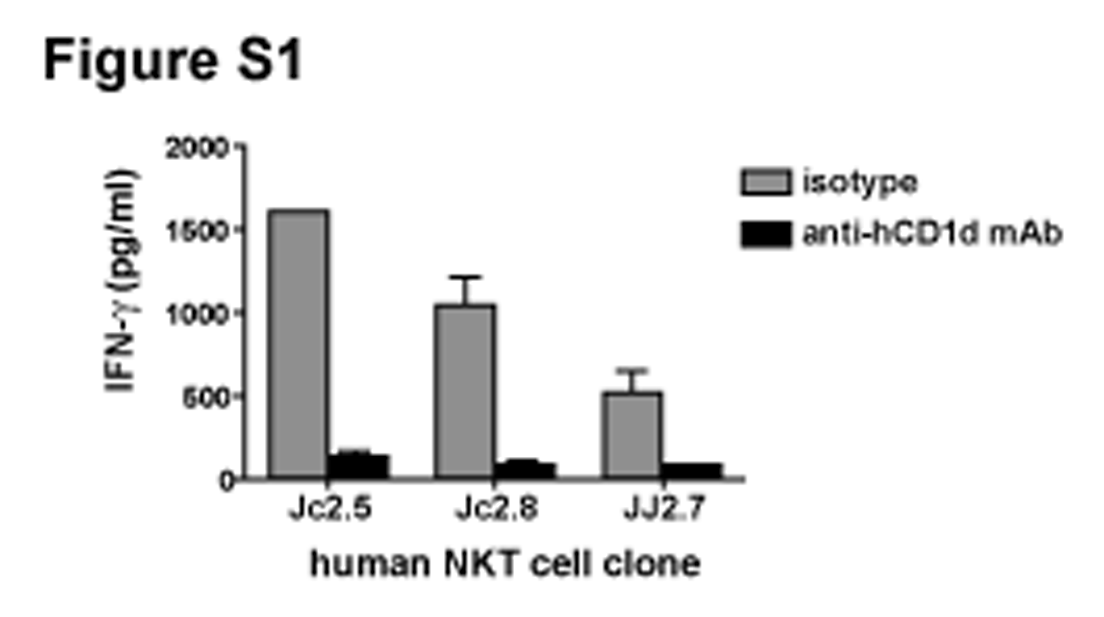

Supplement: Figure S1 — Cytokine production from previously established human iNKT cell clones (Jc25., Jc2.8, JJ2.7) in response to spleen cells from an engrafted mouse treated with α-GalCer is blocked by addition of the CD1d42 anti-human CD1d monoclonal antibody, but not by an isotype-matched negative control antibody. (TIF) [file pone.0021701.s001.tif]

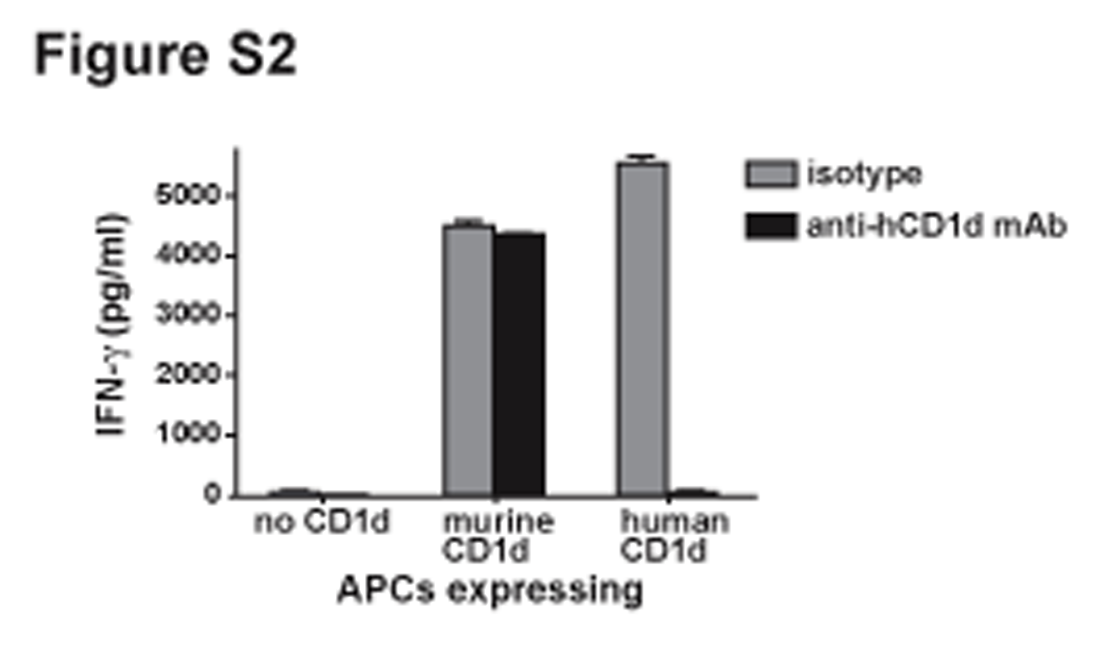

Supplement: Figure S2 — The CD1d42 antibody blocks iNKT cell responses to human but not murine CD1d. Untransfected P815 cells (no CD1d), P815 cells transfected with murine CD1d, and P815 cells transfected with human CD1d were treated with α-GalCer and incubated with a human iNKT cell clone (Jc2.5) in the presence of the anti-CD1d antibody CD1d42 (black bars) or an isotype control mAb (grey bars). (TIF) [file pone.0021701.s002.tif]

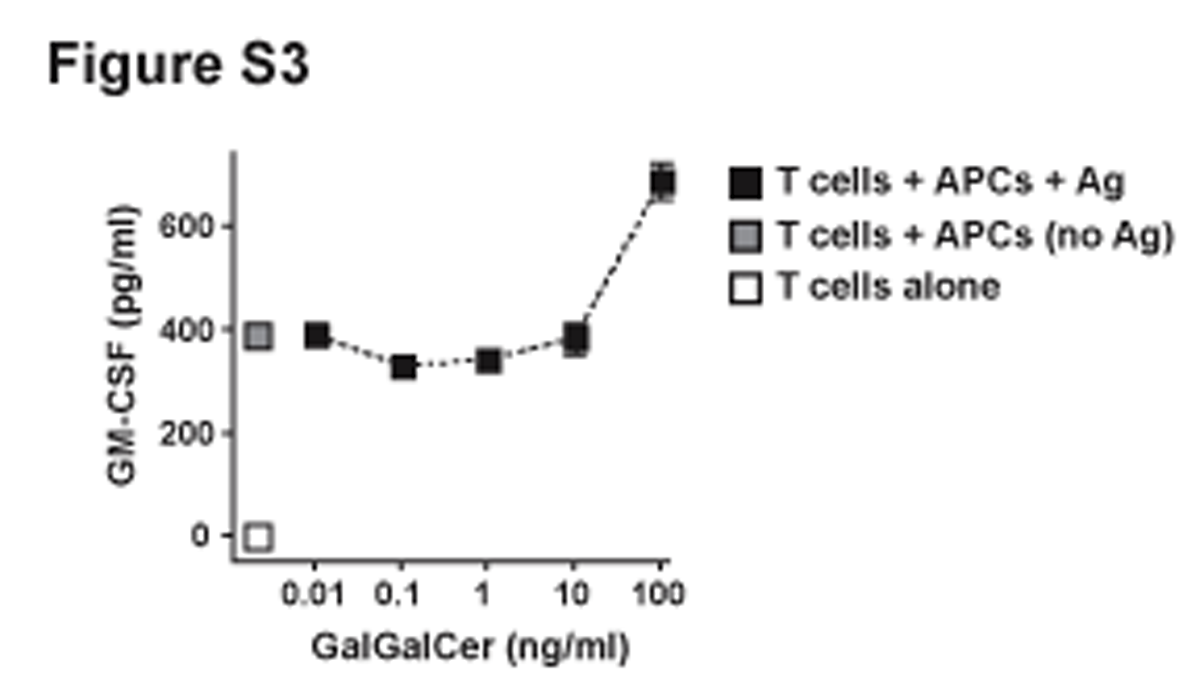

Supplement: Figure S3 — CD1d-mediated antigen presentation by liver cells from engrafted mice was tested using the human iNKT cell clone J24L.17 and a glycolipid called GalGalCer, which requires glycosidic cleavage of the terminal galactose sugar in order to be recognized by iNKT cells. (TIF) [file pone.0021701.s003.tif]

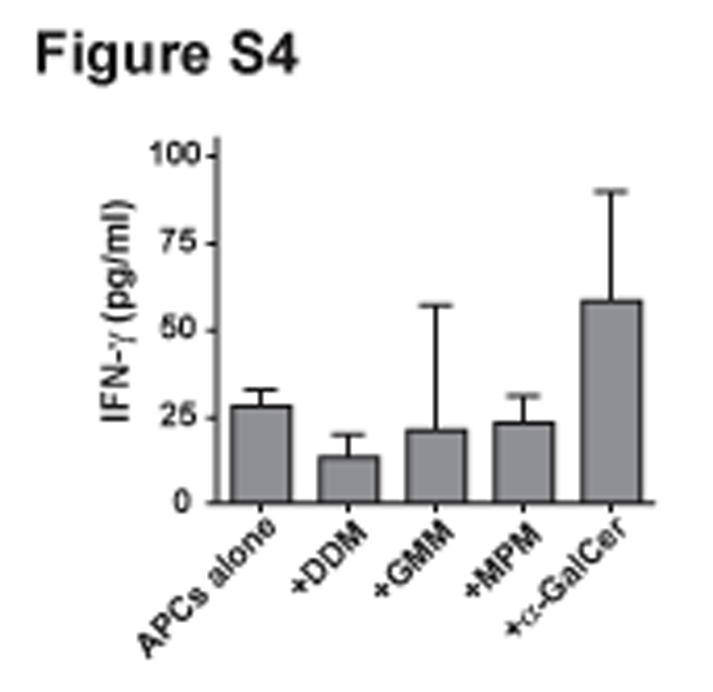

Supplement: Figure S4 — Splenocytes from an engrafted mouse show no significant responses to antigens used for evaluation of CD1-mediated antigen presentation to human T cell lines. (TIF) [file pone.0021701.s004.tif]
